# Supplementary material for: Synergistic biodegradation of aromatic-aliphatic copolyester plastic by a marine microbial consortium
Source: Nat Commun. 2020 Nov 13;11:5790. doi: 10.1038/s41467-020-19583-2 (PMC7666164; doi:10.1038/s41467-020-19583-2)
Supplement: Supplementary file 1 — Supplementary Information [file 41467_2020_19583_MOESM1_ESM.pdf]

# Synergistic biodegradation of aromatic-aliphatic copolyester plastic by a marine microbial consortium

Supplementary information, includes: Supplementary figures 1-14, Supplementary tables 1-5

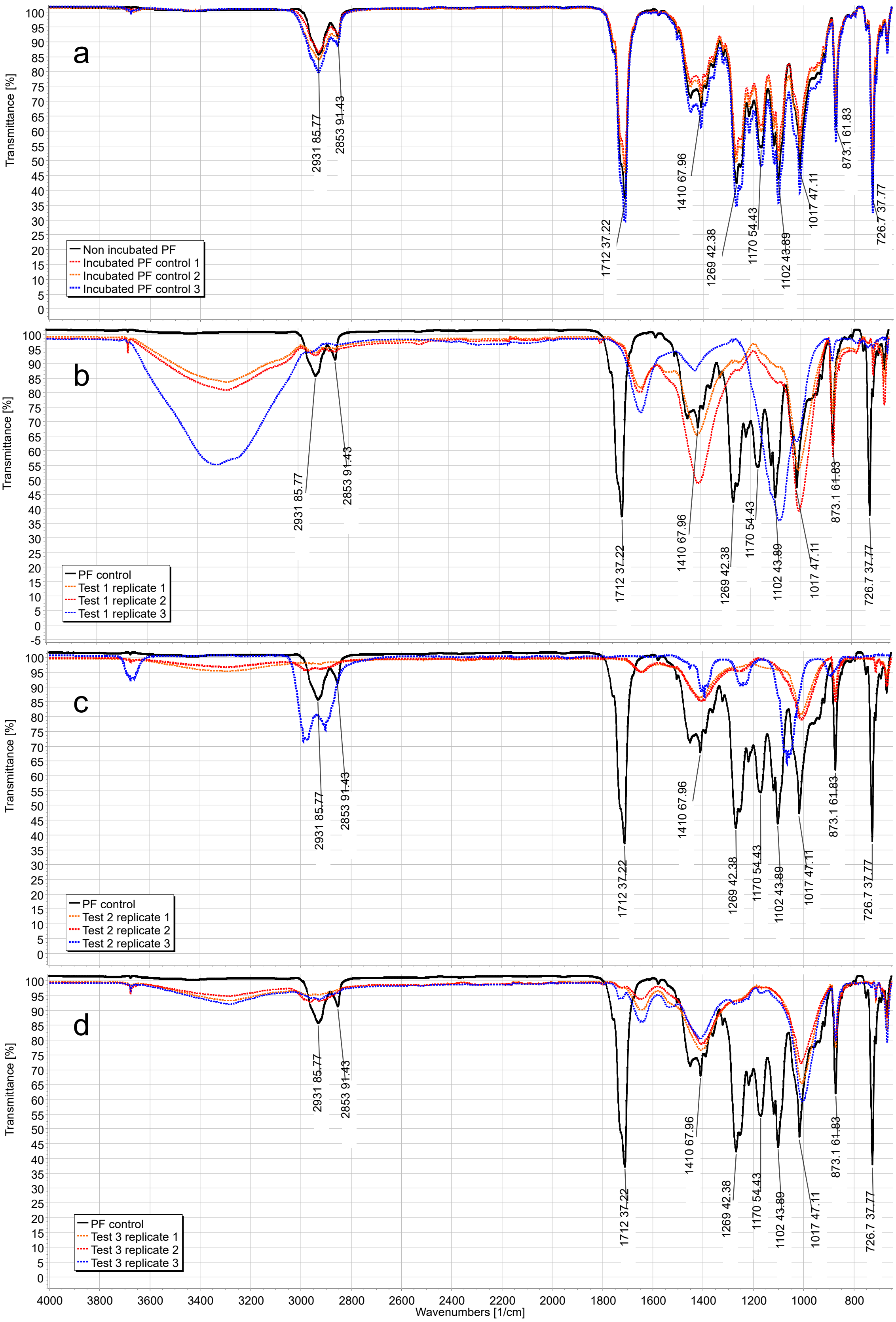
a
b
c
d
Transmittance [%]
Wavenumbers [1/cm]
Non incubated PF
Incubated PF control 1
Incubated PF control 2
Incubated PF control 3
PF control
Test 1 replicate 1
Test 1 replicate 2
Test 1 replicate 3
Test 2 replicate 1
Test 2 replicate 2
Test 2 replicate 3
Test 3 replicate 1
Test 3 replicate 2
Test 3 replicate 3
2931 85.77
2853 91.43
1712 37.22
1410 67.96
1269 42.38
1170 54.43
1102 43.89
1017 47.11
873.1 61.83
726.7 37.77
4000
3800
3600
3400
3200
3000
2800
2600
2400
2200
2000
1800
1600
1400
1200
1000
800

**Figure S1: Fourier transform infrared spectroscopy (FTIR) spectrum of degraded PF.**(a) Untreated PF with corresponding peak values (black spectra) and three replicates (dashed lines of red, orange and blue spectra) of non-inoculated samples are shown. Below, three independent tests (b, c, d) consisting of three biological replicates (dashed lines of red, orange and blue spectra) for biodegraded PF are shown and compared to the untreated PF (black spectra). Remaining solid $CaCO_3$ peaks at 1410, 872 and 712 $cm^{-1}$ were observed in all samples, which was used as inorganic filler in the PF. A broad peak at 3600-3200 $cm^{-1}$ representing an O-H bond was attributed to biofilm exopolysaccharides [1, 2]. Similarly, the C=O-stretch at 1640 $cm^{-1}$ present in all samples possibly originated from amides/proteins originating from the biofilm. The strong peak at 1010 $cm^{-1}$ potentially represents C-O-C or C-O-P stretching vibrations of biofilm-associated polysaccharides. The region between 3000 and 2800 $cm^{-1}$ representing C-H stretch can originate from $-CH_3$ and $>-CH_2$ functional groups of membrane fatty acids [3, 4], however, it cannot be distinguished from the same functional groups present in PF.

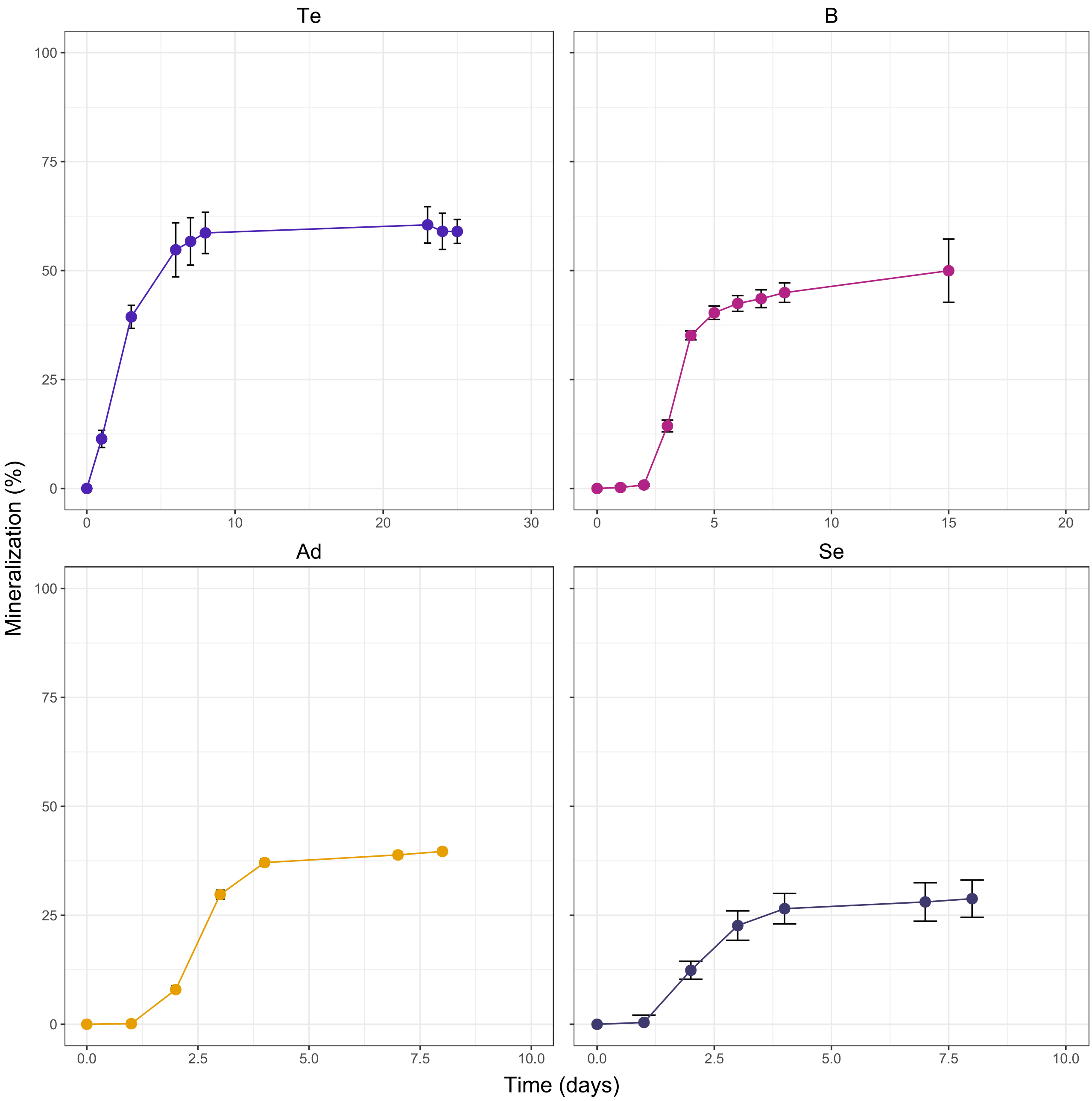


**Figure S2: Mineralization of PF monomers.** $CO_2$ production during I1 growth with terephthalic acid (Te), 1,4-butanediol (B), adipic acid (Ad) or sebacic acid (Se) as sole carbon sources. Each curve is the mean of three biological replicates and error bars represent the standard deviation of these replicates. Source data are provided as a Source Data file.

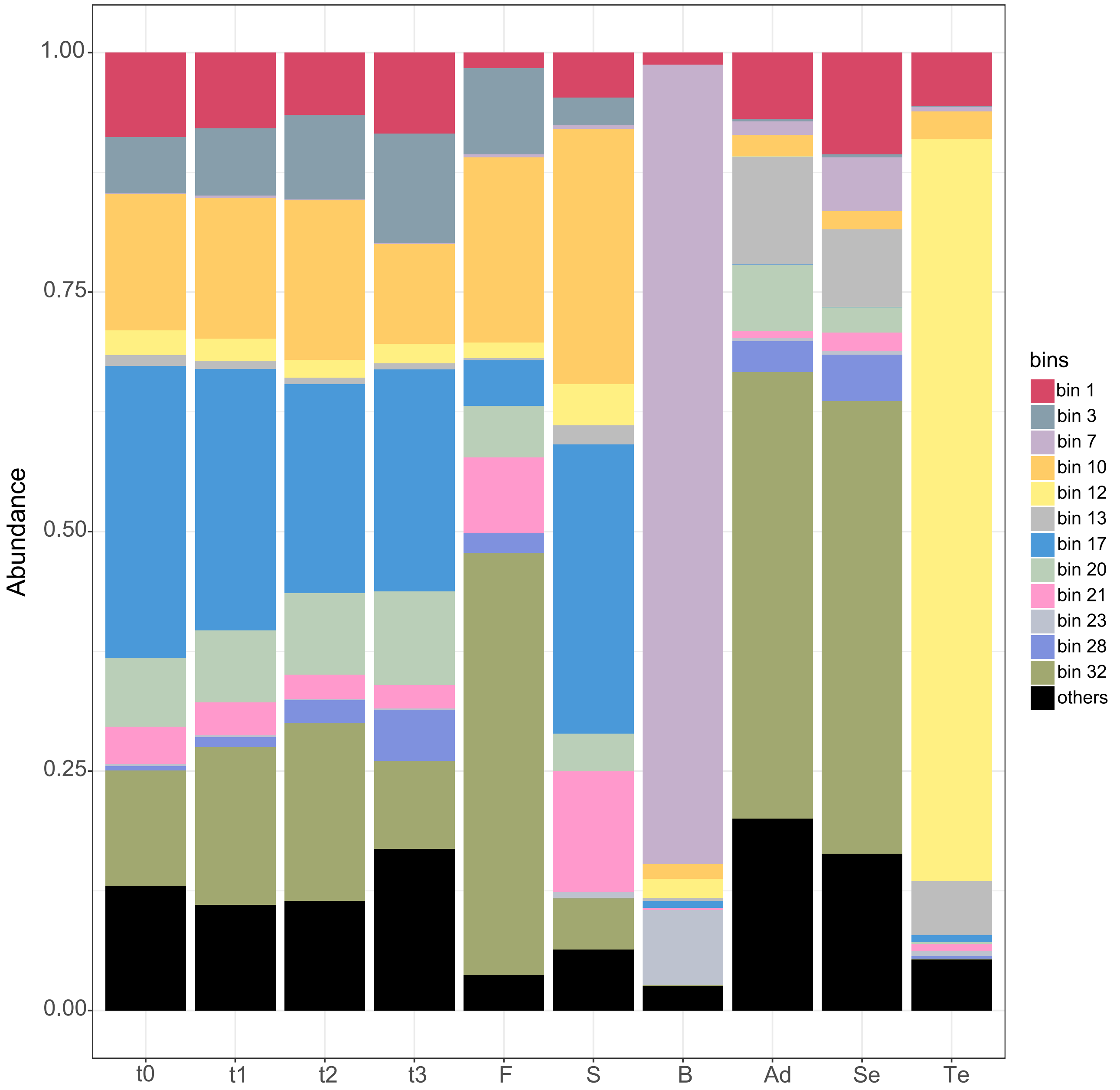


**Figure S3: Relative abundances of the top bins.** Relative abundances of bins $\geq 5\%$ abundant in any condition: during different time points (t0, t1, t2, t3), on film-attached (F), free-living (S), and in the presence of adipic acid (Ad), 1,4-butanediol (B), sebacic acid (Se) and terephthalic acid (Te) as sole carbon source. Each bin is represented by a different color. Source data are provided as a Source Data file.

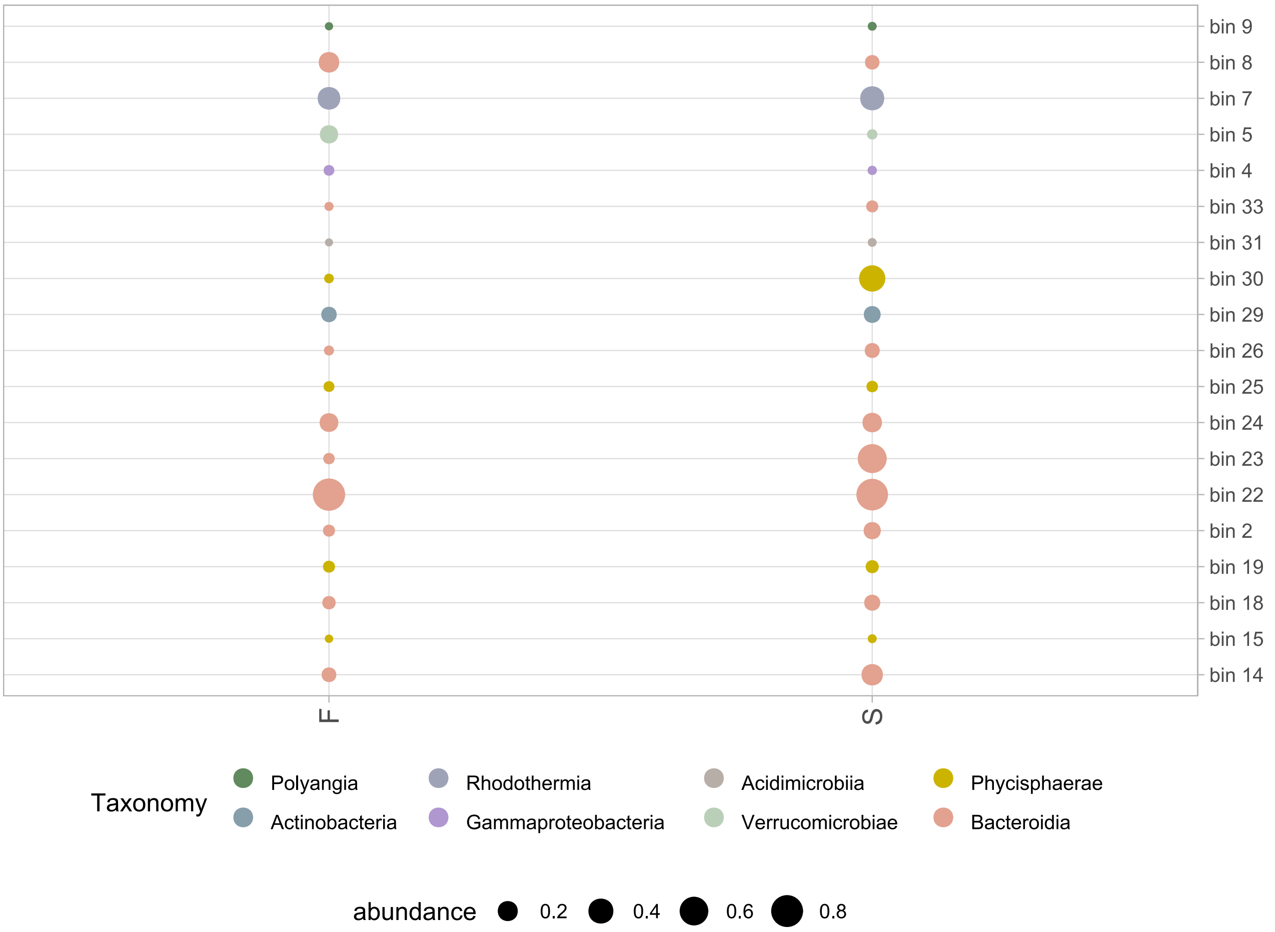


**Figure S4: Relative abundances of the low-abundance bins.** Relative abundances of bins with less than %1 abundance in the binned population in the film-attached (F) and free-living (S) communities. The taxa of each bin is represented by different color. The size of the bubble represents the abundance of each bin. Source data are provided as a Source Data file.

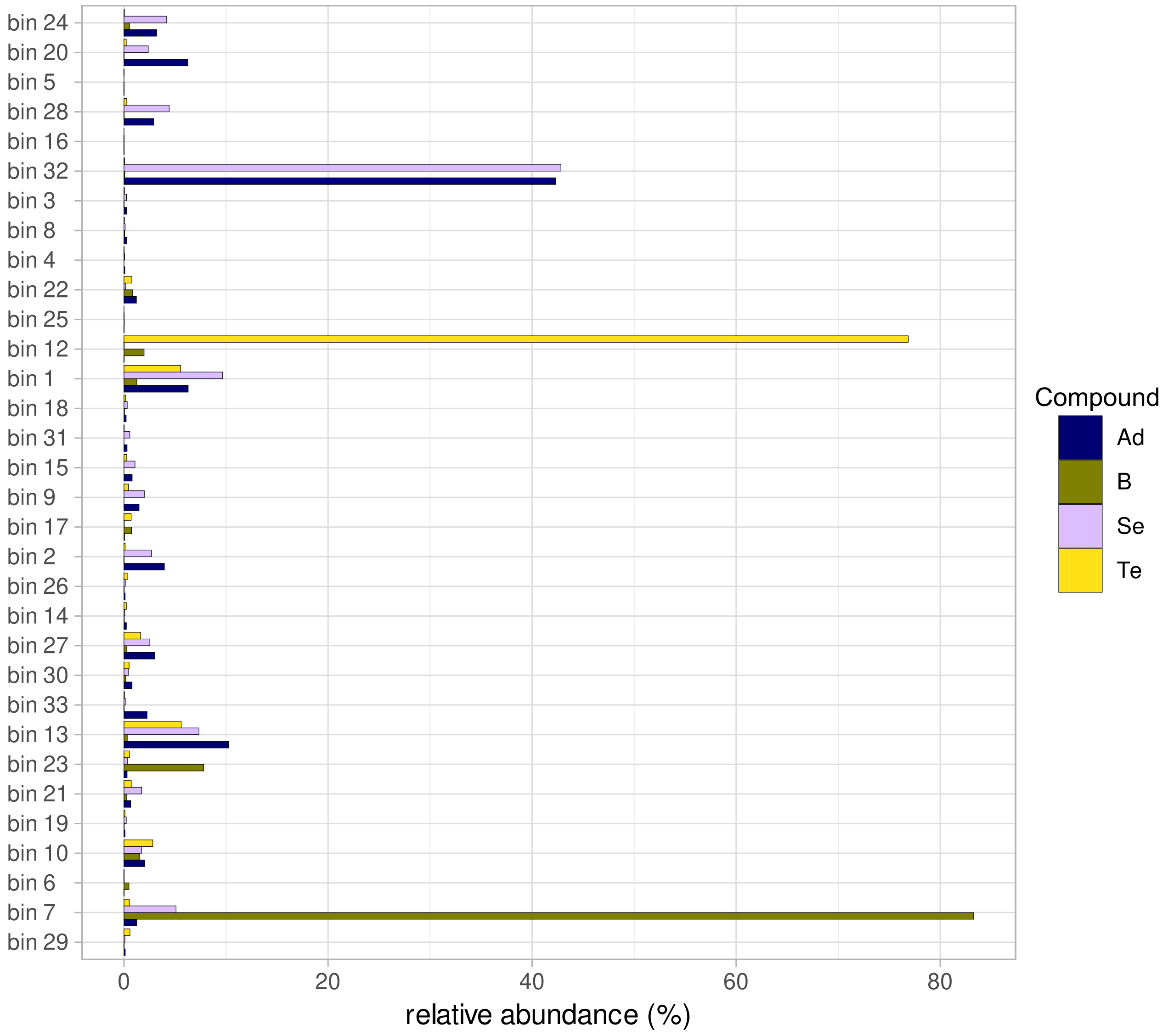


**Figure S5: Relative abundances of the bins in the I1 community growing on monomers.** Abundance of each bin in the binned metagenome when the culture is given each plastic monomer as the only C source: Adipic acid (Ad, dark blue), 1,4-butanediol (B, brown), sebacic acid (Se, pink) and terephthalic acid (Te, yellow). Source data are provided as a Source Data file.

a

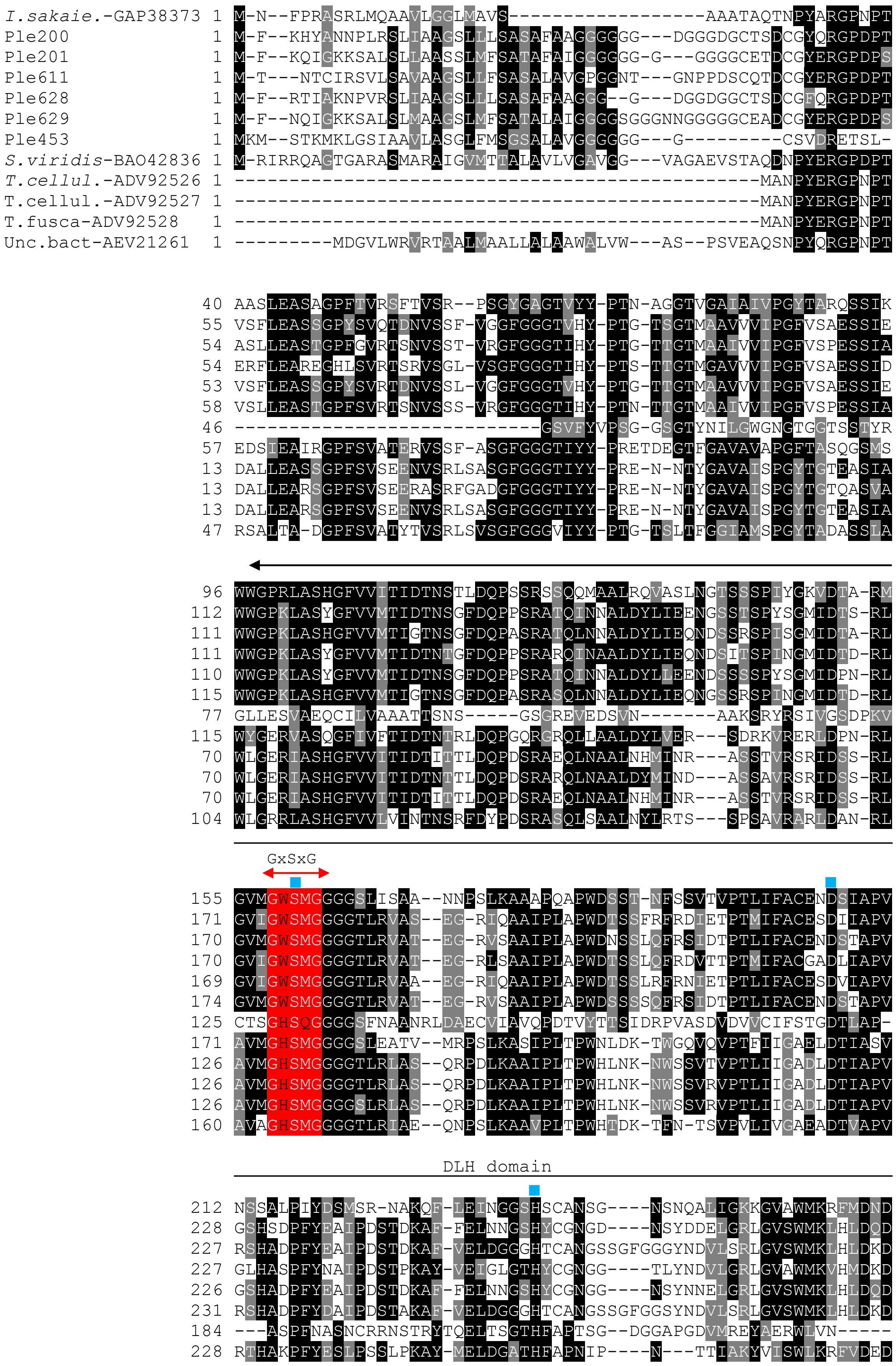

I.sakaie.-GAP38373
Ple200
Ple201
Ple611
Ple628
Ple629
Ple453
S.viridis-BAO42836
T.cellul.-ADV92526
T.cellul.-ADV92527
T.fusca-ADV92528
Unc.bact-AEV21261
GxSxG
DLH domain

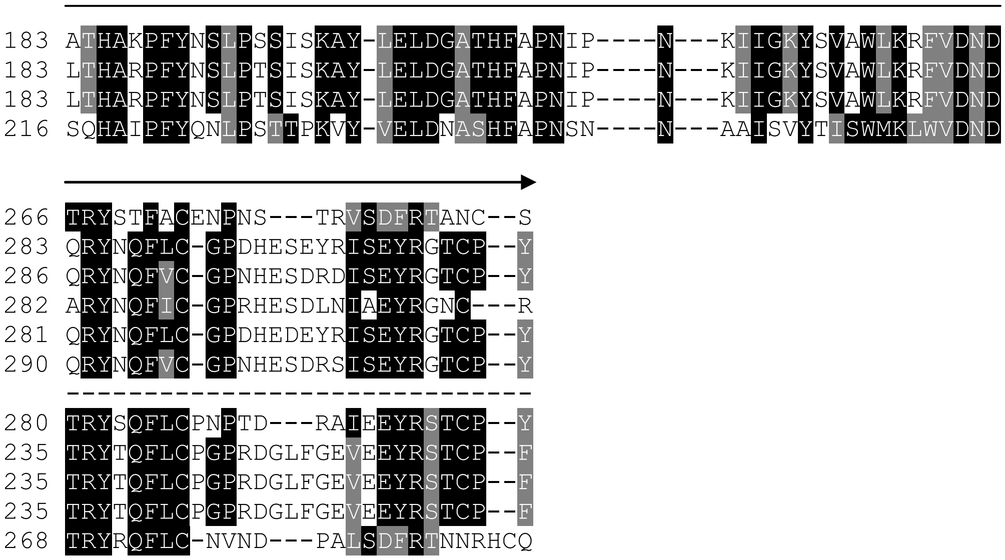
183 ATHAKPFYNSLPSSISKAY-LELDGATHFAPNIP----N---KIIGKYSVAWLKRFVDND
183 LTHARPFYNSLPTSISKAY-LELDGATHFAPNIP----N---KIIGKYSVAWLKRFVDND
183 LTHARPFYNSLPTSISKAY-LELDGATHFAPNIP----N---KIIGKYSVAWLKRFVDND
216 SQHAIPFYQNLPSTTPKVY-VELDNASHFAPNSN----N---AAISVYTISWMKLWVDND
266 TRYSTFACENPNS---TRVSDFRTANC--S
283 QRYNQFLC-GPDHESEYRISEYRGTCP--Y
286 QRYNQFVC-GPNHESDRDISEYRGTCP--Y
282 ARYNQFIC-GPRHESDLNIAEYRGNC---R
281 QRYNQFLC-GPDHEDEYRISEYRGTCP--Y
290 QRYNQFVC-GPNHESDRSISEYRGTCP--Y
280 TRYSQFLCPNPTD---RAIEEYRSTCP--Y
235 TRYTQFLCPGPRDGLFGEVEEYRSTCP--F
235 TRYTQFLCPGPRDGLFGEVEEYRSTCP--F
235 TRYTQFLCPGPRDGLFGEVEEYRSTCP--F
268 TRYRQFLC-NVND---PALSDFRTNNRHCQ

b

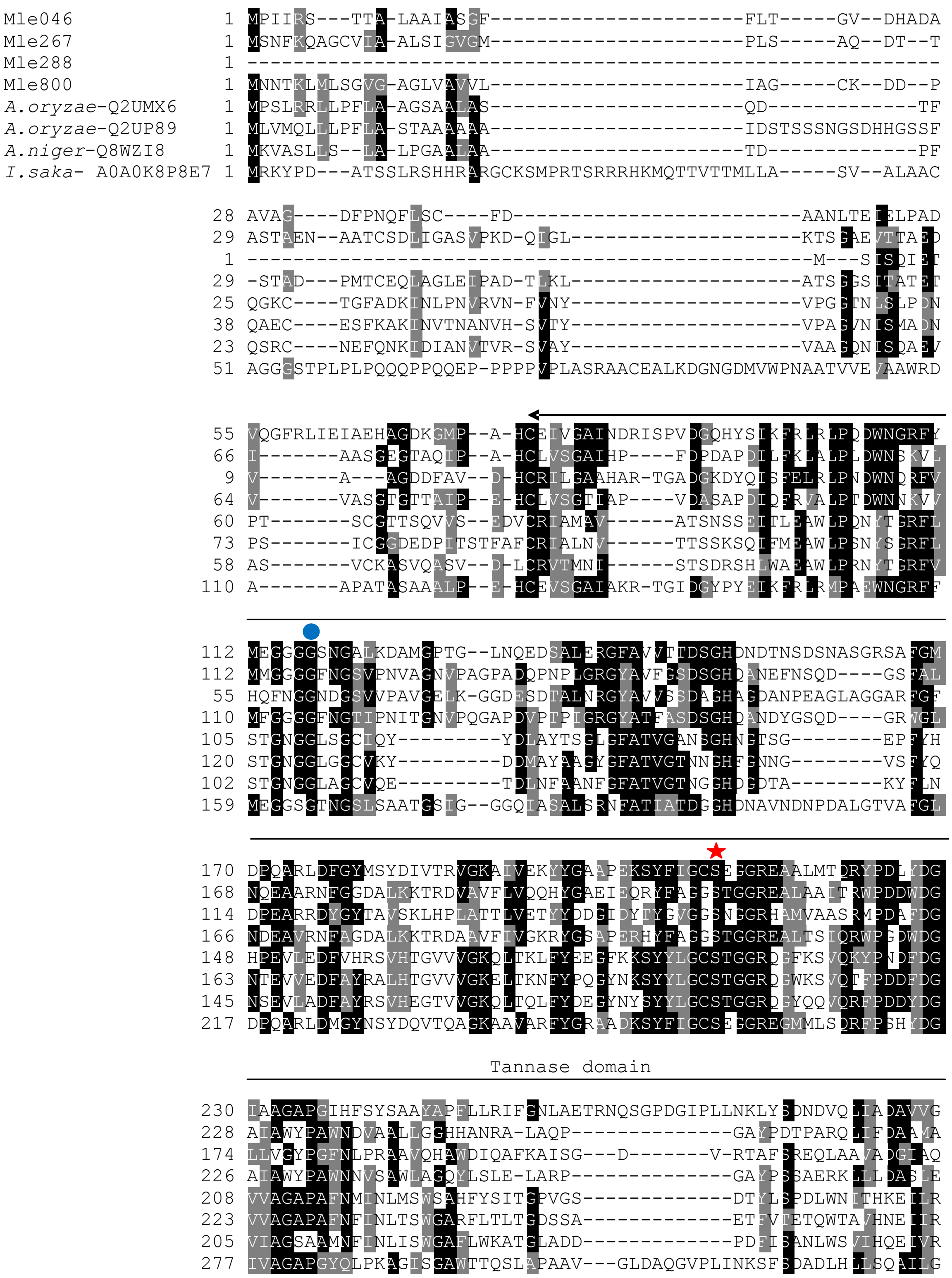

Mle046 1 MPIIRS---TTA-LAAIASGF----------------------FLT-----GV--DHADA
Mle267 1 MSNFKQAGCVIA-ALSIGVGM----------------------PLS-----AQ--DT--T
Mle288 1 ------------------------------------------------------------
Mle800 1 MNNTKLMLSGVG-AGLVAVVL----------------------IAG-----CK--DD--P
A.oryzae-Q2UMX6 1 MPSLRRLLPFLA-AGSAALAS----------------------QD-------------TF
A.oryzae-Q2UP89 1 MLVMQLLLPFLA-STAAAAAA----------------------IDSTSSSNGSDHHGSSF
A.niger-Q8WZI8 1 MKVASLLS--LA-LPGAALAA----------------------TD-------------PF
I.saka- A0A0K8P8E7 1 MRKYPD---ATSSLRSHHRARGCKSMPRTSRRRHKMQTTVTTMLLA-----SV--ALAAC
28 AVAG----DFPNQFLSC----FD-------------------------AANLTEIELPAD
29 ASTAEN--AATCSDLIGASVPKD-QIGL--------------------KTSGAEVTTAED
1 -------------------------------------------------M---SISQIET
29 -STAD---PMTCEQLAGLEIPAD-TLKL--------------------ATSGGSITATET
25 QGKC----TGFADKINLPNVRVN-FVNY--------------------VPGGTNLSLPDN
38 QAEC----ESFKAKINVTNANVH-SVTY--------------------VPAGVNISMADN
23 QSRC----NEFQNKIDIANVTVR-SVAY--------------------VAAGQNISQAEV
51 AGGGSTPLPLPQQQPPQQEP-PPPPVPLASRAACEALKDGNGDMVWPNAATVVEVAAWRD
55 VQGFRLIEIAEHAGDKGMP--A-HCEIVGAINDRISPVDGQHYSIKFRLRLPQDWNGRFY
66 I-------AASGEGTAQIP--A-HCLVSGAIHP----FDPDAPDILFKLALPLDWNSKVL
9 V-------A---AGDDFAV--D-HCRILGAAHAR-TGADGKDYQISFELRLPNDWNQRFV
64 V-------VASGTGTTAIP--E-HCLVSGTIAP----VDASAPDIQFRVALPTDWNNKVV
60 PT-------SCGTTSQVVS--EDVCRIAMAV------ATSNSSEITLEAWLPQNYTGRFL
73 PS-------ICGGDEDPITSTFAFCRIALNV------TTSSKSQIFMEAWLPSNYSGRFL
58 AS-------VCKASVQASV--D-LCRVTMNI------STSDRSHLWAEAWLPRNYTGRFV
110 A-------APATASAAALP--E-HCEVSGAIAKR-TGIDGYPYEIKFRLRMPAEWNGRFF
112 MEGGGGSNGALKDAMGPTG--LNQEDSALERGFAVVTTDSGHDNDTNSDSNASGRSAFGM
112 MMGGGGFNGSVPNVAGNVPAGPADQPNPLGRGYAVFGSDSGHQANEFNSQD----GSFAL
55 HQFNGGNDGSVVPAVGELK-GGDESDTALNRGYAVVSSDAGHAGDANPEAGLAGGARFGF
110 MFGGGGFNGTIPNITGNVPQGAPDVPTPIGRGYATFASDSGHQANDYGSQD----GRWGL
105 STGNGGLSGCIQY---------YDLAYTSGLGFATVGANSGHNGTSG--------EPFYH
120 STGNGGLGGCVKY---------DDMAYAAGYGFATVGTNNGHFGNNG--------VSFYQ
102 STGNGGLAGCVQE---------TDLNFAANFGFATVGTNGGHDGDTA--------KYFLN
159 MEGGSGTNGSLSAATGSIG--GGQIASALSRNFATIATDGGHDNAVNDNPDALGTVAFGL
170 DPQARLDFGYMSYDIVTRVGKAIVEKYYGAAPEKSYFIGCSEGGREAALMTQRYPDLYDG
168 NQEAARNFGGDALKKTRDVAVFLVQQHYGAEIEQRYFAGGSTGGREALAAITRWPDDWDG
114 DPEARRDYGYTAVSKLHPLATTLVETYYDDGIDYTYGVGGSNGGRHAMVAASRMPDAFDG
166 NDEAVRNFAGDALKKTRDAAVFIVGKRYGSAPERHYFAGGSTGGREALTSIQRWPGDWDG
148 HPEVLEDFVHRSVHTGVVVGKQLTKLFYEEGFKKSYYLGCSTGGRQGFKSVQKYPNDFDG
163 NTEVVEDFAYRALHTGVVVGKELTKNFYPQGYNKSYYLGCSTGGRQGWKSVQTFPDDFDG
145 NSEVLADFAYRSVHEGTVVGKQLTQLFYDEGYNYSYYLGCSTGGRQGYQQVQRFPDDYDG
217 DPQARLDMGYNSYDQVTQAGKAAVARFYGRAADKSYFIGCSEGGREGMMLSQRFPSHYDG
Tannase domain
230 IAAGAPGIHFSYSAAYAPFLLRIFGNLAETRNQSGPDGIPLLNKLYSDNDVQLIADAVVG
228 AIAWYPAWNDVAALLGGHHANRA-LAQP--------------GAYPDTPARQLIFDAAMA
174 LLVGYPGFNLPRAAVQHAWDIQAFKAISG---D-------V-RTAFSREQLAAVADGIAQ
226 AIAWYPAWNNVSAWLAGQYLSLE-LARP--------------GAYPSSAERKLLLDASLE
208 VVAGAPAFNMINLMSWSAHFYSITGPVGS-------------DTYLSPDLWNITHKEILR
223 VVAGAPAFNFINLTSWGARFLTLTGDSSA-------------ETFVIETQWTAVHNEIIR
205 VIAGSAAMNFINLISWGAFLWKATGLADD-------------PDFISANLWSVIHQEIVR
277 IVAGAPGYQLPKAGISGAWTTQSLAPAAV---GLDAQGVPLINKSFSDADLHLLSQAILG

```
290 ACDALDGLEDRMSNNIEACTTVTV------LPRLRALTCSGAKEE---GCLLEDQIDAFV
273 ECDGLDGAQDGLISNQTLCNATFDPRTT--QMNGKPVMCQEGQTAAD-ACLTDAQITALE
223 ACDSLDGLEDGYIADTDACQSAFD---------IEALQCDAAITG---HCLSKAQVAALT
271 ACDNLDGLEDGLISDQRQCNDIFDPATA--TLAGVPVRCPGGADDGD-HCLSDAQITALK
255 QCDGIDGAEDGIIEDPSLCSPVLE-----------AIICKPGQNTTE--CLTGKQAHTVR
270 QCDSLDGAKDGIIEDPDLCQPIIE-----------ALLCNATQSSTSGTCLTGAQVKTVN
252 QCDLVDGALDGIIEDPDFCAPVIE-----------RLICDGTTNGTS--CITGAQAAKVN
334 TCDALDGLADGIVDNYRACQAAFDPATAANPANGQALQCVGAKTA---DCLSPVQVTAIK

341 AGMAGPV-----TSDGT--RLYPGHPWDPGIGGRIGDSVNDGFRSWWFGSYDSDQNNARK
330 VMNQGARFNFPLASGETQYPGYNVVWGSDLGLTNR-------DH-------AVQPIITF-L
271 AIHNGPV-----NSQGV--PLYSDWPWDIGIGSG-------DWRFWKLETPIPPWNNQPL
328 AMEAGVSFNFDLASGETGYPGSNVWGADLGITSI-------NT--------PVQPIVTF-L
302 EIFSPLY-----GVNGT--LLYPRMQPGSEVMA---------SSIMYNGQPFQY------
319 GVFSATY-----GLNGS--FLYPRMQPGSELAA---------YSSYYSGTPFAY------
299 RALSDFY-----GPDGT--VYYPRLNYGGEADS---------ASLYFTGSMYSR------
391 RAMAGPV-----NSAGT--PLYNRWAWDAGMSGLSGTTYNQGWRSWWLGSFNSSANNAQR

394 VT-LSTPQHAMLWQTPPVPL--RPDEYVRFEMNF--NIDETPALAYATTDLYPVSSAELG
375 TLGTTQPAMPMERSAPYISV--LLDQWIKYSVTRDPNFDTLSLDAENPG-AWGTRISELS
317 IGVMGSASLAQLFTTPPTEVAGTPQALEQYLLDF--DFDSDAPTIYATTDRYPESAMDYM
373 ALGTSQPAIPMPRNAPYLSV--LVDQWVRYVVARDADFNSFDLDPIDPAPAFAERISELS
340 ------------------------SADWYRYVVYENPNWDATKFSVRDAAVALKQNPFNLQ
357 ------------------------AEDWYRYVVFNNTNWDVATWTVQDAAIANAQDPYQIS
337 ------------------------TEEWYKYVVYNDTNWNSSQWTLESAKLALEQNPFNIQ
444 VSGFSARSWLVDFATPPEPM--PMTQVAARMMKF--DFDIDPLKIWATSGQFTQSSMDWH

449 ---NADSPDLSDFASRGGKLVIYHGAADAAFSALDTIKYWNAVNETADG--Q-AADFARL
432 TL-LDTRVDLDQFHENGGKLLLAHGLSDVLVSTRATQQYYLRLQARMDP--EVVDEFVRY
375 TPPDSENPTLADFNDQGGKMLIFHGVSDPVFSVNDTHWYRKLDANNNG--Q-AEAFARY
431 GP-FNAGTDISAFRDGGGKLLLAHGLSDILVSSRATEIYYQRLLEQMGE--DTVDSFVRY
377 ----TWDADISSFRKAGGKVLTYHGLMDQLISSENSKLYYARVAETMNVPPEELDEFYRF
394 ----TWNGDLSPFQKKGGKVLHYHGMEDAIISSESSKVYYKHVADTMNLSPSELDSFYRF
374 ----AFDPNITAFRDRGGKLLSYHGTQDPIISSTDSKLYYRRVANALNAAPSELDEFYRF
500 ---GATSTDLAAFRDRGGKMILYHGMSDAAFSALDTADYYERLGAAMPG----AAGFARL

503 FIIPGMNHCQGGPATD----------D----VDLLTPLMAWVEDDLPIERLEATV--SNP
489 YEVPGYGHAVSTDFNA----------A----WDSLTALENWREDDTPPADQIVT------
432 YRVPGMPHGAGGPTVD----------D----FDFFTALVDWVEQGKAPEAVVAGVMPGND
488 YEIPGFGHAASSHFNA----------T----WDSLTALENWVERDIAPENQVTM------
433 FQISGMAHCSGGDGAYGIGNQLVTYNDANPENNVLMAMVQWVEKGIAPETIRGAKFTN--
450 FPISGMAHCANADGPSAIGQGTGTFAGNNPQDNVLLAMVQWVEEGVAPDFVRGAKL----
430 FQISGMGHCGDGTGASYIGQGYGTYTSKAPQVNLLRTMVDWVENGKAPEYMPGNKL-N--
553 FLVPGMNHCSGGPGTD----------R----FDMLTPLVAWVERGEAPDQISAWS--GTP

547 DYFGG-KNLSRPLCPYPLYAEYDGEGDPSSAESFTCVAN-
529 DTIKV-PGRTRPMCDYPGWPKYIGTGSLDDAASFVCA--H
478 DAMAQLGQVERKLCPYPQVARYTGG-DDASAASFACE--E
528 DTVGT-PGRSRPLCDYPAWPRYVDGGDPDTAASFECA--E
491 GTGSA-VEYTRKHCRYPRRNVYKGPGNYTDENAWQCV---
506 -NGST-VEYRRKHCKYPKRNRYVGPGSYTDENAWECV---
487 ANGS--IEYMRKHCRYPKHNIHTGPGNYTDPNSWTCV---
597 GYFGV-AARTRPLCPYPQIARYKGSGDINTEANFACAAPP
```

**Figure S6: Multiple alignment of Ple and Mle proteins.** Shaded background represents the similarity of aa residues between the different protein sequences. Among the Ple sequences (a), the aa residues form a catalytic triad (top blue box) and the GXSXG motif (shaded in red) are illustrated. Among the Mle sequences (b), the catalytic triad (top red star) and the oxyanion hole (top blue circle) are shown. Identified conserved domains were indicated with residue-spanning arrows. For the Ple alignment, three additional proteins with known PETase activity were included to those described in the manuscript for a more comprehensive comparison. For the Mle alignment, the IsMHETase and three additional tannase/feruloyl esterase group proteins were used.

a

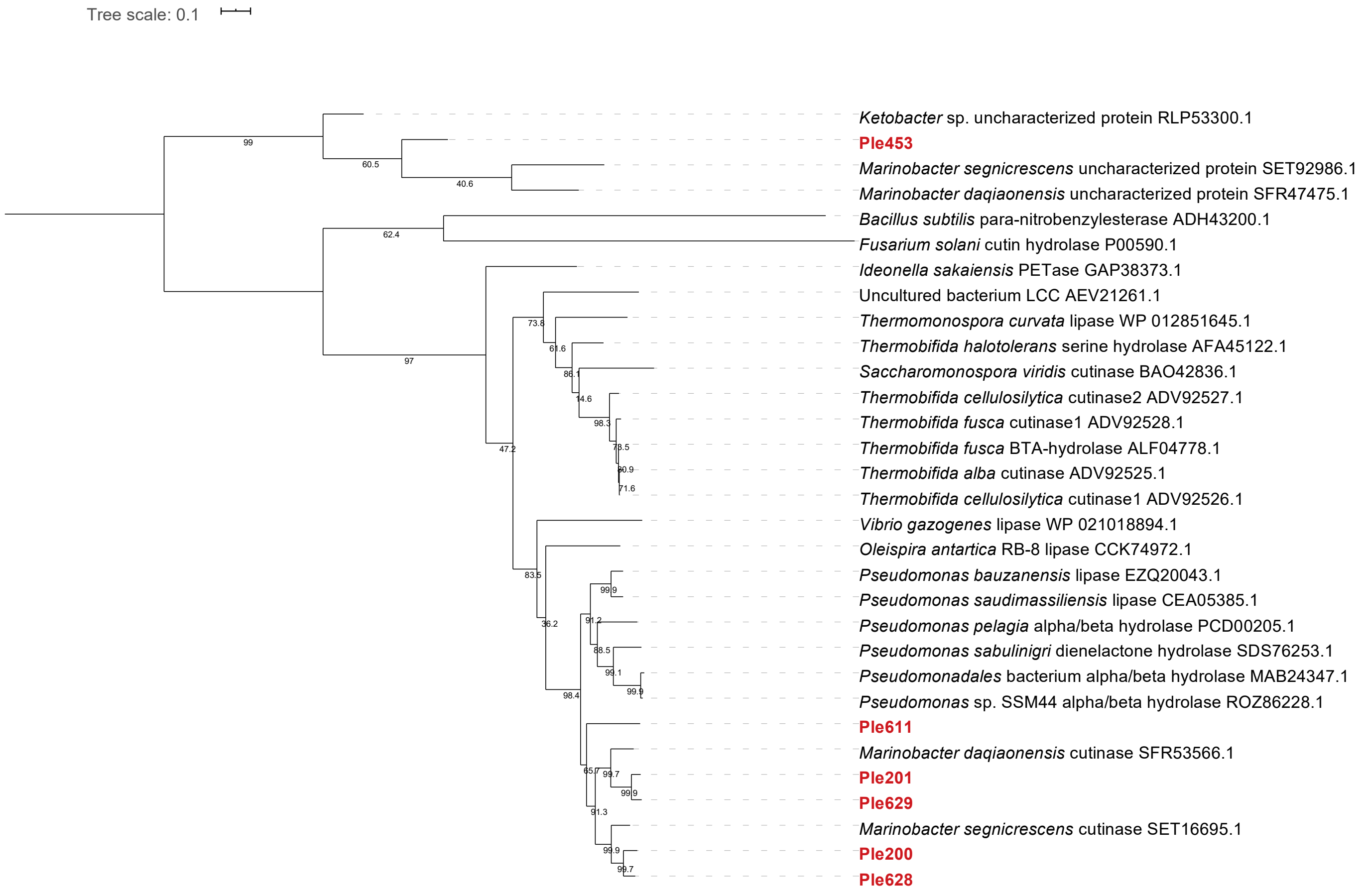

Tree scale: 0.1
Ketobacter sp. uncharacterized protein RLP53300.1
Ple453
Marinobacter segnicrescens uncharacterized protein SET92986.1
Marinobacter daqiaonensis uncharacterized protein SFR47475.1
Bacillus subtilis para-nitrobenzylesterase ADH43200.1
Fusarium solani cutin hydrolase P00590.1
Ideonella sakaiensis PETase GAP38373.1
Uncultured bacterium LCC AEV21261.1
Thermomonospora curvata lipase WP 012851645.1
Thermobifida halotolerans serine hydrolase AFA45122.1
Saccharomonospora viridis cutinase BAO42836.1
Thermobifida cellulosilytica cutinase2 ADV92527.1
Thermobifida fusca cutinase1 ADV92528.1
Thermobifida fusca BTA-hydrolase ALF04778.1
Thermobifida alba cutinase ADV92525.1
Thermobifida cellulosilytica cutinase1 ADV92526.1
Vibrio gazogenes lipase WP 021018894.1
Oleispira antartica RB-8 lipase CCK74972.1
Pseudomonas bauzanensis lipase EZQ20043.1
Pseudomonas saudimassiliensis lipase CEA05385.1
Pseudomonas pelagia alpha/beta hydrolase PCD00205.1
Pseudomonas sabulinigri dienelactone hydrolase SDS76253.1
Pseudomonadales bacterium alpha/beta hydrolase MAB24347.1
Pseudomonas sp. SSM44 alpha/beta hydrolase ROZ86228.1
Ple611
Marinobacter daqiaonensis cutinase SFR53566.1
Ple201
Ple629
Marinobacter segnicrescens cutinase SET16695.1
Ple200
Ple628

b

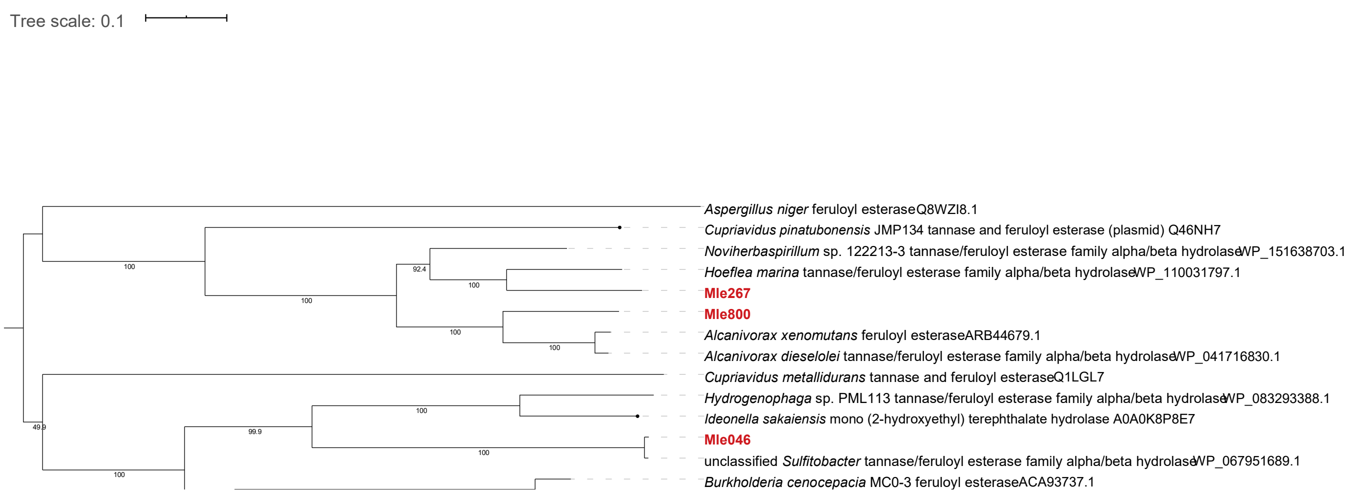


c

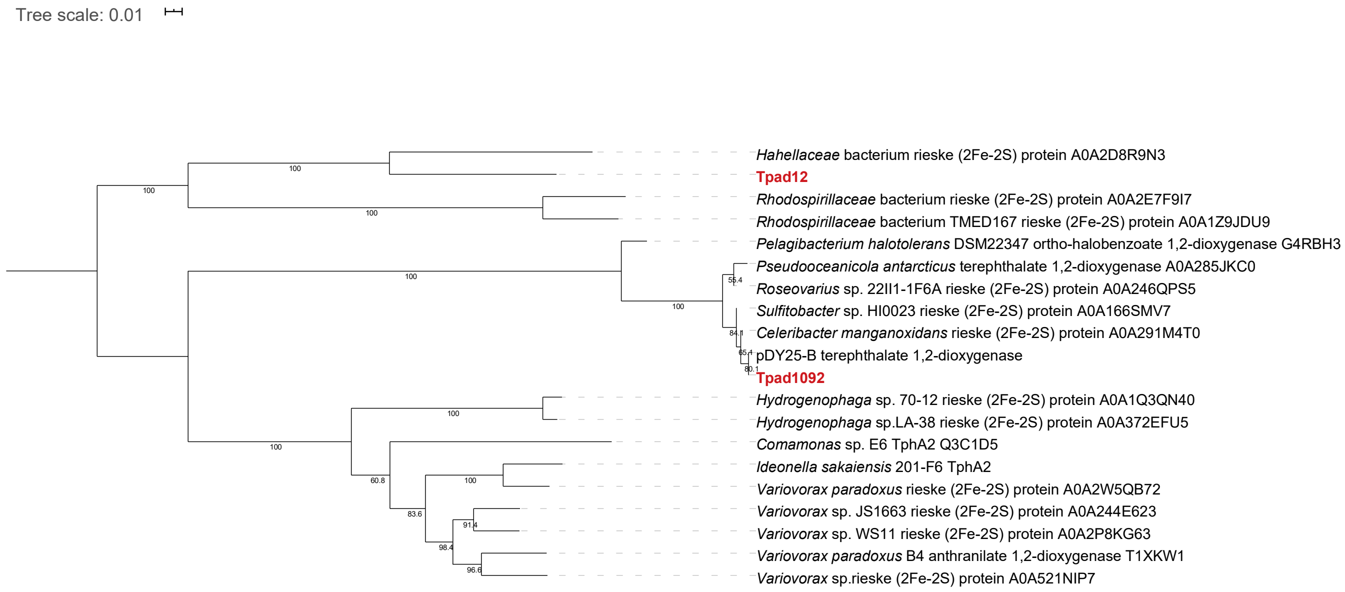


**Figure S7: Phylogenetic classification of Ple, Mle, and TPADO.** Neighbour-joining trees of Ples (a), Mles (b) and TPADO α-subunits (c). Sequences originating in this study are shown in red. The scale represents aa substitution per site.

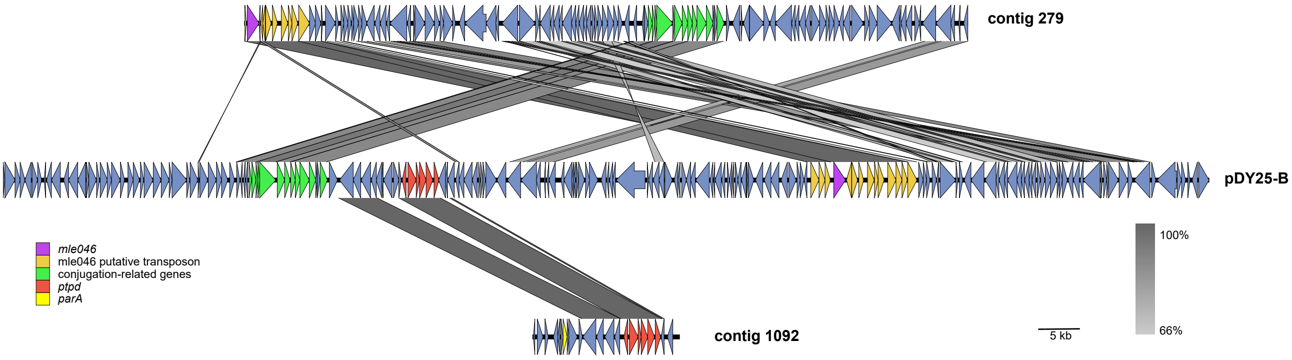


**Figure S8: Nucleotide comparison of contig 279, pDYB25-B and contig 1092.** CDS are represented by arrows. Catabolic, horizontal transfer-related, and conjugation-related genes are indicated by the colour key featured below the alignment. The aligned regions are shaded in grey according to their nucleotide sequence similarity.

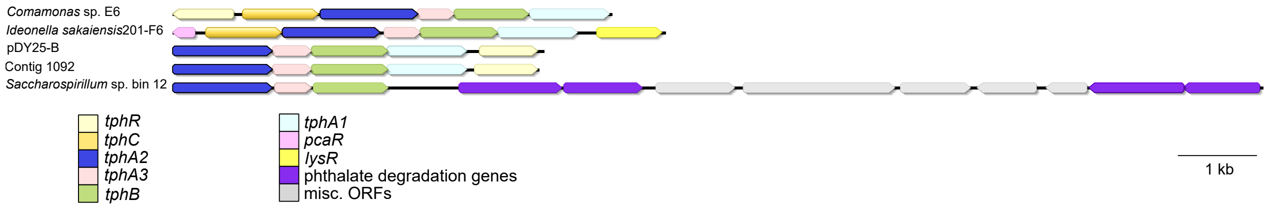


**Figure S9: TPD operon comparison.** The TPD cluster of contig 1092 and bin 12 are compared to strains carrying known TPD genes. CDS are represented by arrows. The genes that encode a terephthalate regulator (*tphR*) (light yellow), terephthalate permease (*tphC*) (yellow), terephtalate dioxygenase (*tphA2A3*) (blue and light pink respectively), terephthalate dihydrodiol dehydrogenase (*tphB*) (light green), reductase component (*tphA1*) (light blue), phtalate degradation cluster *pht* (purple) as well as putative *pcaR* (pink) and *lysR*-type regulators (bright yellow) are shown.

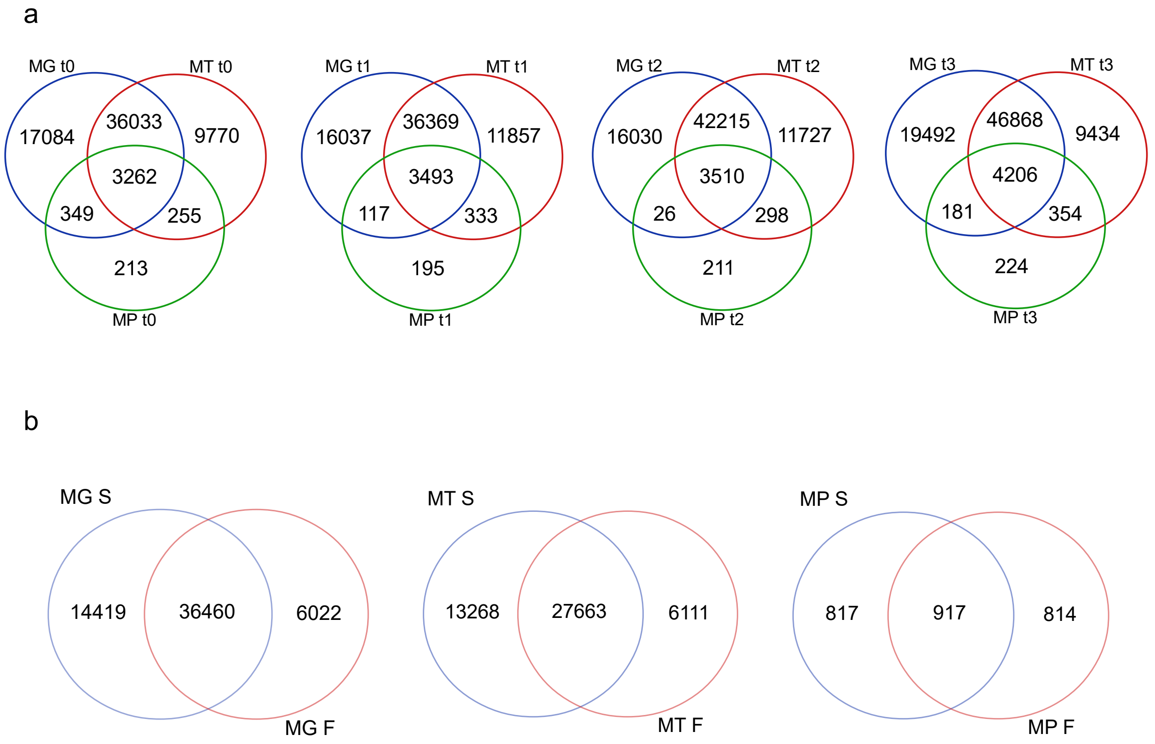


**Figure S10: Comparison of gene, transcript and protein abundances.** Venn diagrams of gene (MG), transcript (MT) and protein (MP) counts for each (a) time point (t0, t1, t2, t3) and among (b) the film-attached (F) and free-living bacteria (S).

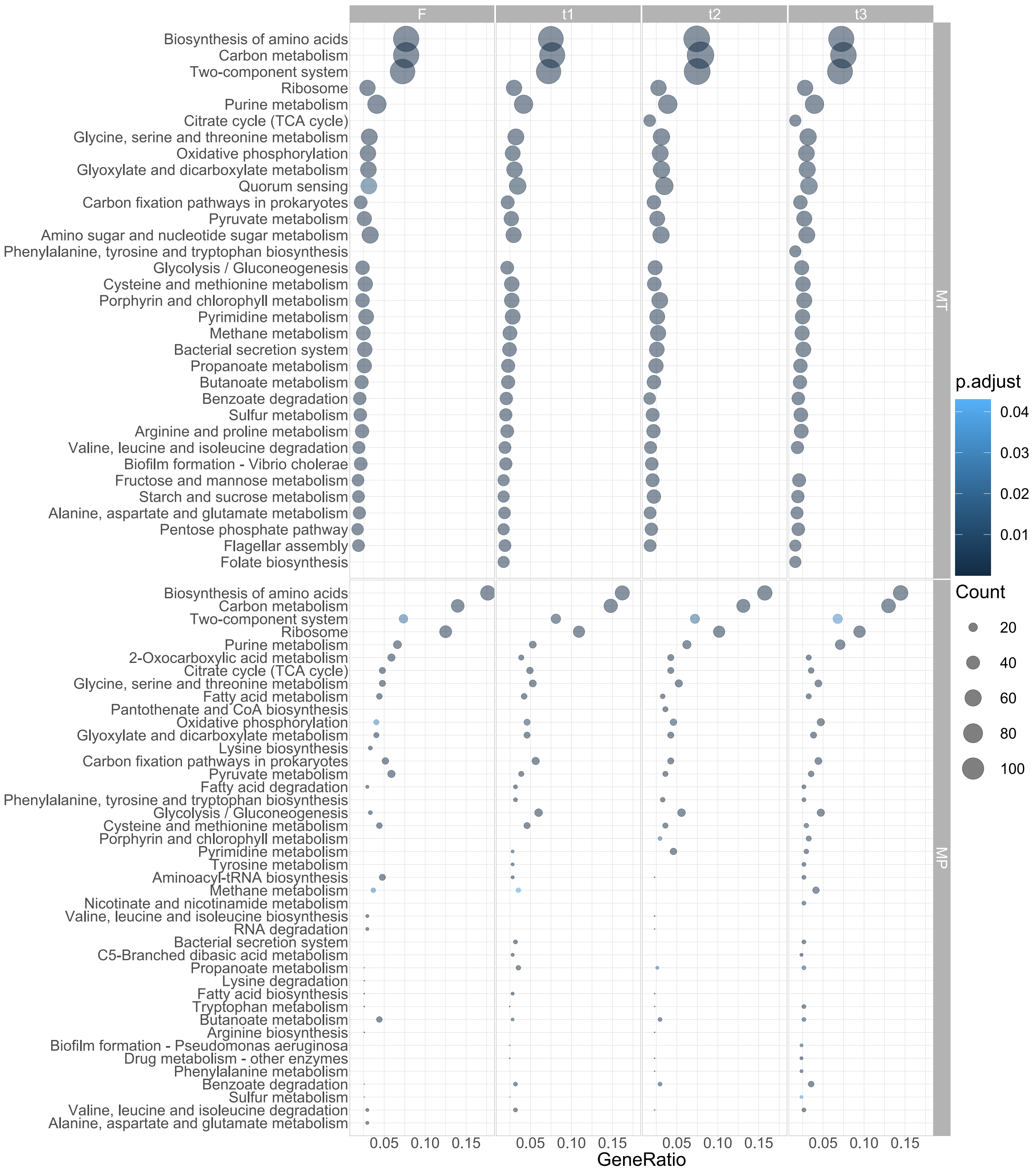
F
t1
t2
t3
MT
MP
Biosynthesis of amino acids
Carbon metabolism
Two-component system
Ribosome
Purine metabolism
Citrate cycle (TCA cycle)
Glycine, serine and threonine metabolism
Oxidative phosphorylation
Glyoxylate and dicarboxylate metabolism
Quorum sensing
Carbon fixation pathways in prokaryotes
Pyruvate metabolism
Amino sugar and nucleotide sugar metabolism
Phenylalanine, tyrosine and tryptophan biosynthesis
Glycolysis / Gluconeogenesis
Cysteine and methionine metabolism
Porphyrin and chlorophyll metabolism
Pyrimidine metabolism
Methane metabolism
Bacterial secretion system
Propanoate metabolism
Butanoate metabolism
Benzoate degradation
Sulfur metabolism
Arginine and proline metabolism
Valine, leucine and isoleucine degradation
Biofilm formation - Vibrio cholerae
Fructose and mannose metabolism
Starch and sucrose metabolism
Alanine, aspartate and glutamate metabolism
Pentose phosphate pathway
Flagellar assembly
Folate biosynthesis
Biosynthesis of amino acids
Carbon metabolism
Two-component system
Ribosome
Purine metabolism
2-Oxocarboxylic acid metabolism
Citrate cycle (TCA cycle)
Glycine, serine and threonine metabolism
Fatty acid metabolism
Pantothenate and CoA biosynthesis
Oxidative phosphorylation
Glyoxylate and dicarboxylate metabolism
Lysine biosynthesis
Carbon fixation pathways in prokaryotes
Pyruvate metabolism
Fatty acid degradation
Phenylalanine, tyrosine and tryptophan biosynthesis
Glycolysis / Gluconeogenesis
Cysteine and methionine metabolism
Porphyrin and chlorophyll metabolism
Pyrimidine metabolism
Tyrosine metabolism
Aminoacyl-tRNA biosynthesis
Methane metabolism
Nicotinate and nicotinamide metabolism
Valine, leucine and isoleucine biosynthesis
RNA degradation
Bacterial secretion system
C5-Branched dibasic acid metabolism
Propanoate metabolism
Lysine degradation
Fatty acid biosynthesis
Tryptophan metabolism
Butanoate metabolism
Arginine biosynthesis
Biofilm formation - Pseudomonas aeruginosa
Drug metabolism - other enzymes
Phenylalanine metabolism
Benzoate degradation
Sulfur metabolism
Valine, leucine and isoleucine degradation
Alanine, aspartate and glutamate metabolism
0.05 0.10 0.15
0.05 0.10 0.15
0.05 0.10 0.15
0.05 0.10 0.15
GeneRatio
p.adjust
0.04
0.03
0.02
0.01
Count
20
40
60
80
100

**Figure S11: KEGG enrichments.** Representation of the top 30 KEGG pathways enriched in the metatranscriptome (MT) and metaproteome (MP) at each time point (t1, t2, t3) and in the film-attached community (F) relative to their controls, this is time point t0 and free-living community respectively. The GeneRatio is defined as the number of genes/proteins identified in the time series and film-attached communities associated to a specific pathway divided by the total number of genes/proteins of the corresponding pathway. Counts of genes/proteins represent the absolute number of genes/proteins associated to a pathway and their values are represented by the size of each bubble. The p-values are adjusted using the Benjamini-Hochberg method (p.adjust) and the cutoff is 0.05. As shown, the significance for most transcripts and proteins were below the significance threshold ($\leq 0.05$).

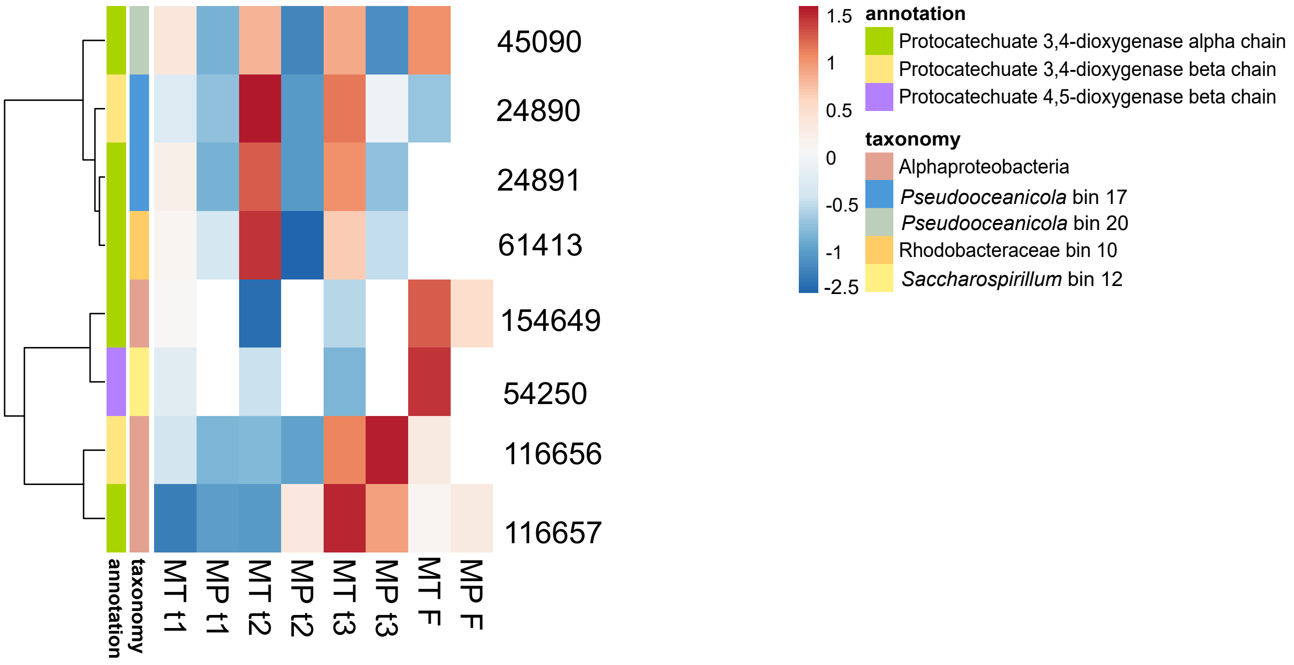


**Figure S12: Heatmap of PCD genes and proteins.** Fold change profiles of PCD genes identified at the metatranscriptome (MT) and metaproteome (MP) levels during different time points t1, t2, t3 and in the film-attached (F) community. The fold changes in (F) equal to the same amount of fold change in the free-living community with the opposite sign.The average of at least two biological replicates were used to calculate fold changes.The scale shows $\log_2$-transformed fold changes. Samples were clustered according to the highest fold-change in the metatranscriptome. The taxonomic classifications for the genes that could not be allocated to a certain bin were assessed from the taxonomic affiliation of the nearest-related BLAST hit. These were all collected under "Alphaproteobacteria". Source data are provided as a Source Data file.

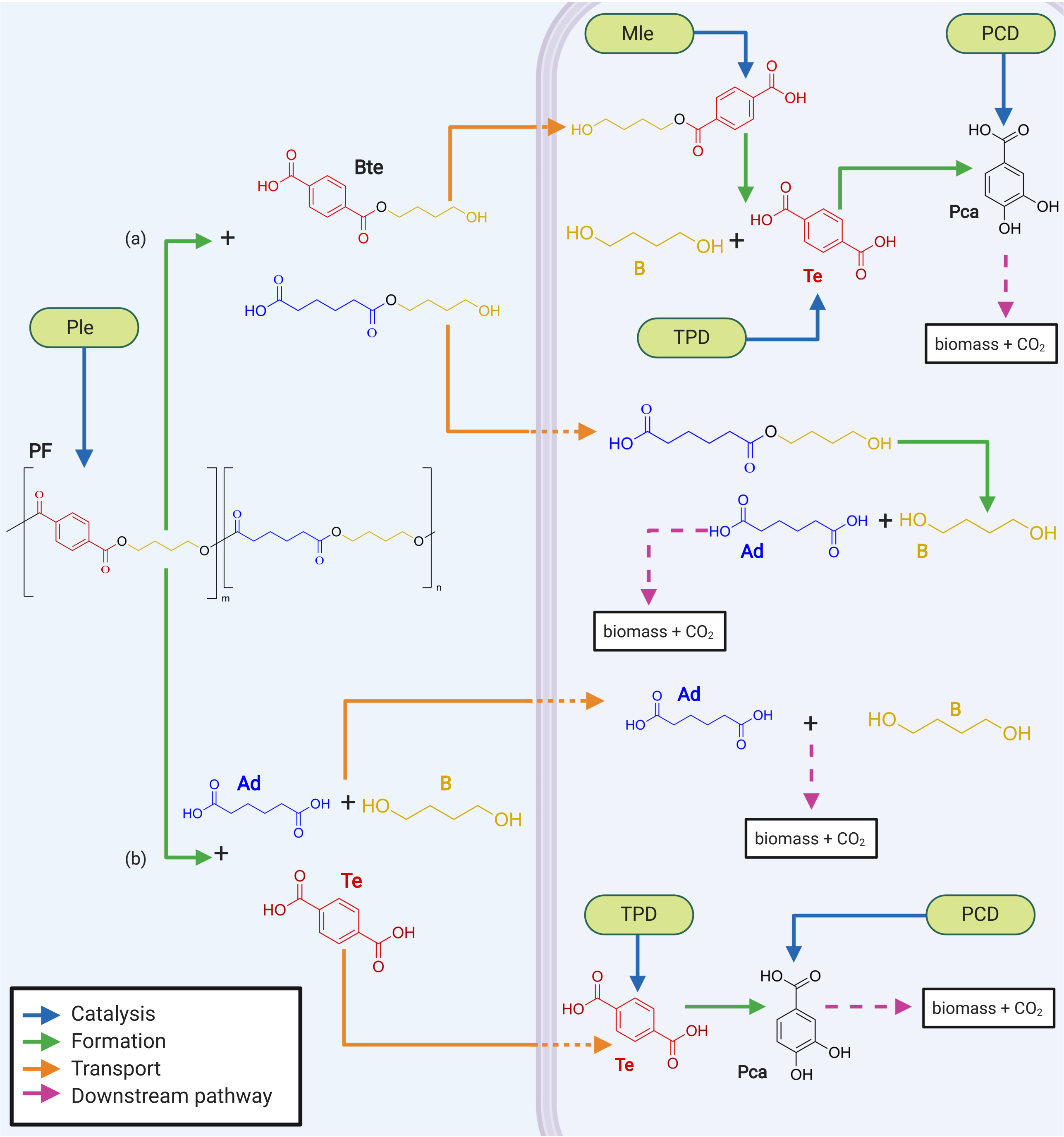


**Figure S13: Alternative routes to PF biodegradation.** Schematic overview of proposed PBAT-based blend film (PF) biodegradation pathways through (a) monoesters and (b) free monomers. Both routes lead to the formation of biomass and $CO_2$. A periplasmatic α/β hydrolase (Ple) hydrolyzes PF to produce monoesters (e.g. terephthalate-butanediol monoester (BTe)) or free monomers (adipic acid (Ad), 1,4-butanediol (B), terephthalic acid (Te)). These are transported to the cells and mineralized via different pathways. BTe is degraded by Mles and Te by TPD enzymes. Te is converted to protocatechuate (Pca), which can be further cleaved by PCA dioxygenases (PCD) (created with Biorender.com).

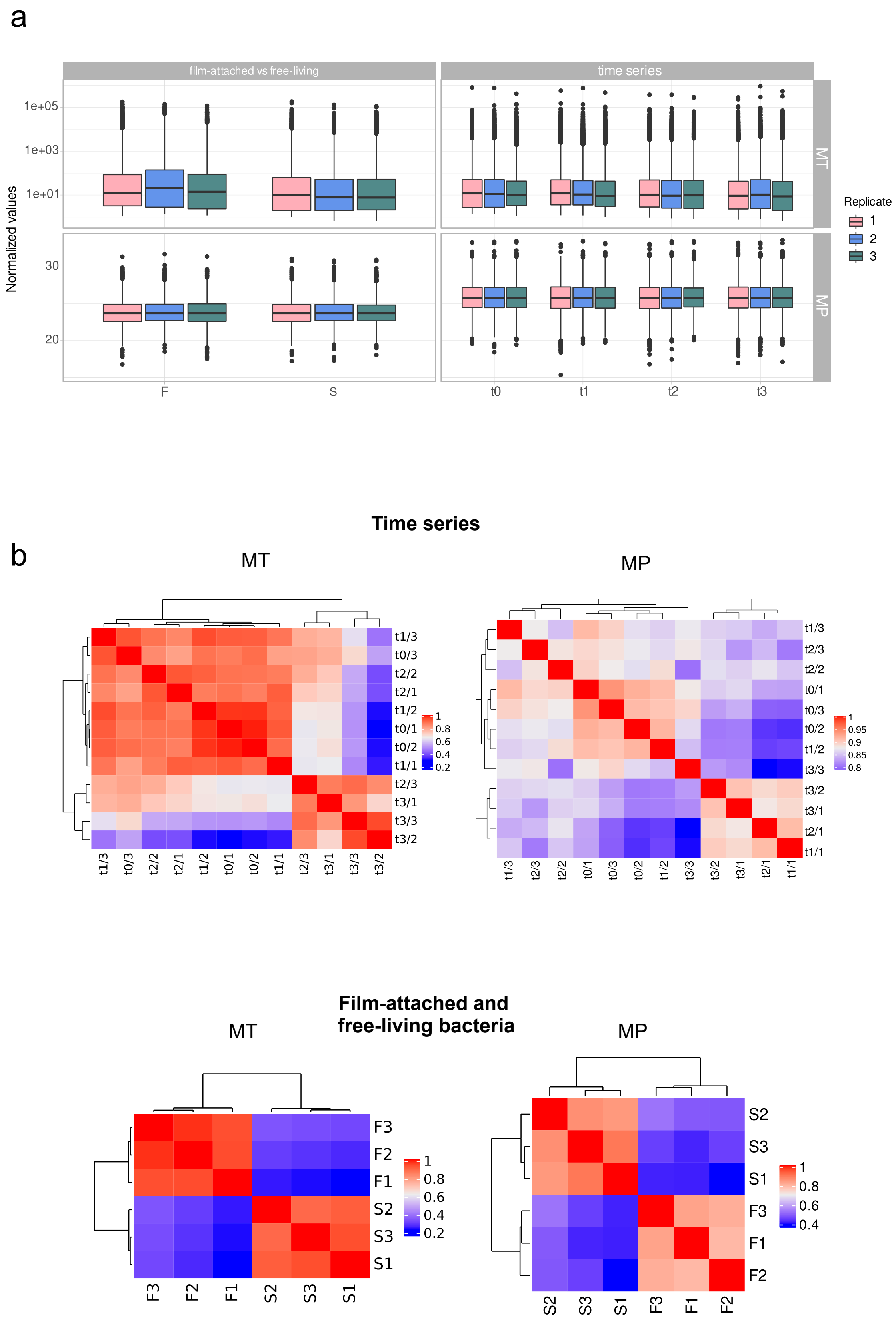
a
film-attached vs free-living
time series
MT
MP
Normalized values
1e+05
1e+03
1e+01
30
20
F
S
t0
t1
t2
t3
Replicate
1
2
3
Time series
b
MT
MP
t1/3
t0/3
t2/2
t2/1
t1/2
t0/1
t0/2
t1/1
t2/3
t3/1
t3/3
t3/2
1
0.8
0.6
0.4
0.2
t2/3
t2/2
t0/1
t0/3
t0/2
t1/2
t3/3
t3/2
t3/1
t2/1
t1/1
0.95
0.9
0.85
0.8
Film-attached and
free-living bacteria
MT
MP
F3
F2
F1
S2
S3
S1
1
0.8
0.6
0.4
0.2
S2
S3
S1
F3
F1
F2
1
0.8
0.6
0.4

**Figure S14: Comparison of the biological replicates for the MT and MP.** (a) The distribution of the normalized transcript (MT) counts and normalized peptide areas (MP) of each biological replicate (1, 2, 3) were compared between film-attached (F) and free-living samples (S) as well as for the time series (t0, t1, t2, t3). Box plots show the 1.5 × interquartile range, the maximum and minimum data point within the range of samples represented by whiskers, the median value of the samples represented by the center line and the outliers of the different data sets ($n = 3$). (b) Unsupervised clustering with Euclidean distance metric of the log-transformed normalized counts (MT) and peptide areas (MP) of each biological replicate (1, 2, 3) among the time series samples (t0, t1, t2, t3) and film-attached (F) and free-living (S) samples after pairwise correlation.

**Table S1: Monomers detection.** The table shows peak areas for adipic acid (Ad), terephthalic acid (Te) and sebacic acid (Se), and the terephthalate-butanediol monoester (BTe) normalized to mg PF. Results are shown for biological triplicates (1,2,3) and for two different time points: after eight days (t1) and after twenty days (t2). ND: not detected

| compound | t1-1 | t1-2 | t1-3 | mean | deviation | t2-1 | t2-2 | t2-3 | mean | deviation |
|---|---|---|---|---|---|---|---|---|---|---|
| Ad | 9986 | 13719 | 5369 | 9691 | 3415 | ND | 198 | 655 | 427 | 229 |
| Te | 49370 | 81824 | 25973 | 52389 | 22901 | 2543 | 449 | 3891 | 2294 | 1416 |
| Se | 4492 | 2187 | 2077 | 2919 | 1114 | ND | ND | ND | ND | ND |
| BTe | 10087 | 9742 | 10811 | 10213 | 446 | ND | ND | ND | ND | ND |

**Table S2:** Genome statistics of the bins. The taxonomy is calculated based on GTDB [5].

| bin | %completeness | %contamination | size (bp) | order | family | genus |
|---|---|---|---|---|---|---|
| bin 1 | 98.17 | 0.57 | 2,685,723 | Flavobacteriales | Flavobacteriaceae | *Aequorivita* |
| bin 2 | 96.22 | 1.868 | 4,563,287 | Rhodobacterales | Rhodobacteraceae | *Maritimibacter* |
| bin 3 | 96.31 | 1.304 | 2,449,326 | Micavibrionales | Micavibrionaceae | UBA2638 |
| bin 4 | 97.43 | 3.418 | 4,758,108 | Microtrichales | Ilumatobacteraceae | *Ilumatobacter* |
| bin 5 | 97.72 | 0 | 2,903,571 | Phycisphaerales | SM1A02 | |
| bin 6 | 97.97 | 0 | 3,036,494 | Actinomycetales | Microbacteriaceae | *Microbacterium* |
| bin 7 | 99.6 | 0.862 | 4,206,910 | Pseudomonadales | Oleiphilaceae | *Marinobacter* |
| bin 8 | 96.68 | 0.978 | 3,442,906 | Rhodobacterales | Rhodobacteraceae | *Roseovarius* |
| bin 9 | 96.12 | 6.311 | 10,246,861 | Polyangiales | Sandaracinaceae | |
| bin 10 | 99.36 | 0.532 | 3,629,643 | Rhodobacterales | Rhodobacteraceae | UBA3069 |
| bin 12 | 99.47 | 1.169 | 4,539,134 | Pseudomonadales | Saccharospirillaceae | *Saccharospirillum* |
| bin 13 | 98.03 | 1.33 | 4,951,164 | Rhodobacterales | Rhodobacteraceae | *Marinovum* |
| bin 14 | 99.95 | 0.204 | 3,444,071 | Rhizobiales | Devosiaceae | *Maritalea* |
| bin 15 | 100 | 0 | 5,708,228 | Cytophagales | Cyclobacteriaceae | *Cyclobacterium* |
| bin 16 | 99.66 | 0 | 2,852,433 | Flavobacteriales | Flavobacteriaceae | *Gelidibacter* |
| bin 17 | 94.52 | 0.025 | 3,094,070 | Rhodobacterales | Rhodobacteraceae | *Pseudooceanicola* |
| bin 18 | 98.47 | 0.869 | 4,779,831 | Thalassobaculales | Thalassobaculaceae | *Thalassobaculum* |
| bin 19 | 99.24 | 0.165 | 3,722,788 | Flavobacteriales | Flavobacteriaceae | *Muricauda* |
| bin 20 | 96.38 | 0.451 | 3,722,696 | Rhodobacterales | Rhodobacteraceae | *Pseudooceanicola* |
| bin 21 | 89.43 | 0.431 | 3,501,129 | Pseudomonadales | Oleiphilaceae | *Marinobacter* |
| bin 22 | 99.72 | 0.775 | 4,016,722 | Rhizobiales | Rhizobiaceae | *Hoeflea* |
| bin 23 | 100 | 0.777 | 4,363,639 | Rhodospirillales | Thalassospiraceae | *Thalassospira* |
| bin 24 | 56.03 | 0 | 3,792,569 | Rhodospirillales | Thalassospiraceae | *Thalassospira* |
| bin 25 | 96.67 | 0.907 | 3,595,020 | Flavobacteriales | Flavobacteriaceae | *Muricauda* |
| bin 26 | 72.98 | 1.241 | 3,320,445 | Rhodobacterales | Rhodobacteraceae | *Maritimibacter* |
| bin 27 | 98.53 | 0.478 | 4,883,485 | Rhodobacterales | Rhodobacteraceae | FREY01 |
| bin 28 | 96.61 | 0.905 | 2,779,823 | Xanthomonadales | Marinicellaceae | |
| bin 29 | 94.94 | 0 | 3,383,596 | Balneolales | Balneolaceae | *Balneola* |
| bin 30 | 99 | 0.541 | 3,424,160 | Flavobacteriales | Flavobacteriaceae | *Muricauda* |
| bin 31 | 100 | 2.947 | 6,658,105 | Verrucomicrobiales | DEV007 | |
| bin 32 | 99.09 | 0.742 | 4,746,010 | Pseudomonadales | Oleiphilaceae | *Marinobacter* |
| bin 33 | 61.84 | 0 | 2,654,327 | Rhodobacterales | Rhodobacteraceae | *Paracoccus* |

**Table S3: Percentage of unbinned reads**.The table shows percentage of unbinned reads per biological replicate (1, 2, 3) for each time point (t0, t1, t2, t3), among the film-attached (F) and free-living communities (S) as well as when the culture received the monomers as C source (adipic acid (Ad), sebacic acid (Se), terephthalic acid (Te) and 1,4-butanediol (B)). The monomer experiments were performed in duplicate.

| t0-1 | t0-2 | t0-3 | t1-1 | t1-2 | t1-3 | t2-1 | t2-2 | t2-3 | t3-1 | t3-2 | t3-3 |
|---|---|---|---|---|---|---|---|---|---|---|---|
| 17.38 | 18.39 | 16.43 | 13.44 | 18.04 | 16.11 | 12.41 | 14.75 | 14.98 | 17.28 | 11.59 | 15.33 |

| F-1 | F-2 | F-3 | S-1 | S-2 | S-3 |
|---|---|---|---|---|---|
| 7.87 | 7.96 | 9.54 | 23.01 | 19.30 | 21.55 |

| Ad-1 | Ad-2 | Se-1 | Se-2 | Te-1 | Te-2 | B-1 | B-2 |
|---|---|---|---|---|---|---|---|
| 4.88 | 4.61 | 16.43 | 17.34 | 15.34 | 14.77 | 8.55 | 8.78 |

**Table S4: Putative PCA degrading genes**. The table shows putative PCA dioxygenase subunits detected in the I1 metagenome, the bins they are located on along with the taxonomy, their nearest relatives (determined by blastP against the nr database), the taxonomic affiliation of the nearest relative, and the % id on the aa level.

| gene id | protein annotation | bins | taxonomy/genus | nearest relative | %id |
|---|---|---|---|---|---|
| MNNIHDME_116656 | Protocatechuate 3,4-dioxygenase beta chain | NA | NA | WP_096705154.1 | 100 |
| MNNIHDME_116657 | Protocatechuate 3,4-dioxygenase alpha chain | NA | NA | WP_076626049.1 | 99.5 |
| MNNIHDME_121399 | Protocatechuate 4,5-dioxygenase alpha chain | bin 2 | *Maritimibacter* | WP_138422719.1 | 95.5 |
| MNNIHDME_121620 | Protocatechuate 4,5-dioxygenase beta chain | NA | NA | WP_161141828.1 | 72.5 |
| MNNIHDME_127017 | Protocatechuate 4,5-dioxygenase alpha chain | NA | NA | WP_175275155.1 | 89.9 |
| MNNIHDME_131226 | Protocatechuate 3,4-dioxygenase alpha chain | NA | NA | WP_093151368.1 | 99 |
| MNNIHDME_131227 | Protocatechuate 3,4-dioxygenase beta chain | NA | NA | WP_028793662.1 | 98.7 |
| MNNIHDME_134955 | Protocatechuate 4,5-dioxygenase alpha chain | NA | NA | NKB54468.1 | 79.5 |
| MNNIHDME_140475 | Protocatechuate 4,5-dioxygenase alpha chain | NA | NA | WP_138422719.1 | 97 |
| MNNIHDME_140628 | Protocatechuate 3,4-dioxygenase beta chain | NA | NA | WP_028287416.1 | 98.8 |
| MNNIHDME_140629 | Protocatechuate 3,4-dioxygenase alpha chain | NA | NA | WP_028287415.1 | 92 |
| MNNIHDME_143140 | Protocatechuate 4,5-dioxygenase beta chain | NA | NA | WP_127556989.1 | 96.9 |
| MNNIHDME_144822 | Protocatechuate 4,5-dioxygenase beta chain | NA | NA | WP_172297030.1 | 90.2 |
| MNNIHDME_154649 | Protocatechuate 3,4-dioxygenase alpha chain | NA | NA | WP_090610495.1 | 98.8 |
| MNNIHDME_155846 | Protocatechuate 3,4-dioxygenase alpha chain | NA | NA | WP_069300576.1 | 89.6 |
| MNNIHDME_23422 | Protocatechuate 4,5-dioxygenase beta chain | bin 12 | *Saccharospirillum* | WP_127556989.1 | 100 |
| MNNIHDME_23423 | Protocatechuate 4,5-dioxygenase alpha chain | bin 12 | *Saccharospirillum* | WP_127556991.1 | 100 |
| MNNIHDME_24890 | Protocatechuate 3,4-dioxygenase beta chain | bin 17 | *Pseudooceanicola* | WP_170848806.1 | 91.1 |
| MNNIHDME_24891 | Protocatechuate 3,4-dioxygenase alpha chain | bin 17 | *Pseudooceanicola* | WP_093446090.1 | 87.9 |
| MNNIHDME_45090 | Protocatechuate 3,4-dioxygenase alpha chain | bin 20 | *Pseudooceanicola* | WP_093446090.1 | 100 |
| MNNIHDME_45091 | Protocatechuate 3,4-dioxygenase beta chain | bin 20 | *Pseudooceanicola* | WP_170848806.1 | 100 |
| MNNIHDME_50411 | Protocatechuate 3,4-dioxygenase beta chain | bin 27 | FREY01 | WP_108799836.1 | 90.5 |
| MNNIHDME_50412 | Protocatechuate 3,4-dioxygenase alpha chain | bin 27 | FREY01 | WP_108799835.1 | 87.4 |
| MNNIHDME_54249 | Protocatechuate 4,5-dioxygenase alpha chain | bin 12 | *Saccharospirillum* | WP_127558406.1 | 100 |
| MNNIHDME_54250 | Protocatechuate 4,5-dioxygenase beta chain | bin 12 | *Saccharospirillum* | WP_127558408.1 | 100 |
| MNNIHDME_59732 | Protocatechuate 4,5-dioxygenase beta chain | bin 2 | *Maritimibacter* | MAS04007.1 | 79.3 |
| MNNIHDME_59733 | Protocatechuate 4,5-dioxygenase alpha chain | bin 2 | *Maritimibacter* | MAS04008.1 | 73.6 |
| MNNIHDME_61412 | Protocatechuate 3,4-dioxygenase beta chain | bin 10 | UBA3069 | WP_043744219.1 | 89.6 |
| MNNIHDME_61413 | Protocatechuate 3,4-dioxygenase alpha chain | bin 10 | UBA3069 | WP_176855484.1 | 84.9 |
| MNNIHDME_76147 | Protocatechuate 3,4-dioxygenase alpha chain | bin 23 | *Thalassospira* | WP_033069880.1 | 99.5 |
| MNNIHDME_76148 | Protocatechuate 3,4-dioxygenase beta chain | bin 23 | *Thalassospira* | WP_033069881.1 | 97.9 |
| MNNIHDME_77534 | Protocatechuate 3,4-dioxygenase alpha chain | bin 18 | *Thalassobaculum* | WP_093151368.1 | 86.5 |
| MNNIHDME_77535 | Protocatechuate 3,4-dioxygenase beta chain | bin 18 | *Thalassobaculum* | WP_028793662.1 | 91.1 |
| MNNIHDME_79080 | Protocatechuate 4,5-dioxygenase beta chain | bin 2 | *Maritimibacter* | WP_008335186.1 | 96.6 |
| MNNIHDME_82611 | Protocatechuate 4,5-dioxygenase beta chain | bin 13 | *Marinovum* | WP_048532237.1 | 91.8 |
| MNNIHDME_82612 | Protocatechuate 4,5-dioxygenase alpha chain | bin 13 | *Marinovum* | WP_048532236.1 | 93.2 |
| MNNIHDME_87636 | Protocatechuate 4,5-dioxygenase beta chain | bin 2 | *Maritimibacter* | MAM61413.1 | 96 |
| MNNIHDME_91531 | Protocatechuate 3,4-dioxygenase beta chain | bin 24 | *Thalassospira* | WP_062954372.1 | 99.1 |
| MNNIHDME_91532 | Protocatechuate 3,4-dioxygenase alpha chain | bin 24 | *Thalassospira* | WP_062954373.1 | 97.9 |
| MNNIHDME_95630 | Protocatechuate 3,4-dioxygenase alpha chain | bin 24 | *Thalassospira* | WP_062953866.1 | 98.1 |
| MNNIHDME_95631 | Protocatechuate 3,4-dioxygenase beta chain | bin 24 | *Thalassospira* | WP_120225792.1 | 99.6 |
| MNNIHDME_96243 | Protocatechuate 4,5-dioxygenase alpha chain | bin 2 | *Maritimibacter* | MAM62849.1 | 92.4 |
| MNNIHDME_96244 | Protocatechuate 4,5-dioxygenase beta chain | bin 2 | *Maritimibacter* | WP_138422724.1 | 95 |
| MNNIHDME_96365 | Protocatechuate 4,5-dioxygenase beta chain | NA | NA | WP_171220659.1 | 78.5 |

**Table S5: Mapping percentage.** The table contains the amount of reads of MG and MT that could be successfully mapped on the combined assembly.The amount of reads are shown per biological replicate (1, 2, 3) for each time point (t0, t1, t2, t3) and among the film-attached (F) and free-living communities (S).

| | MG | MT |
|---|---|---|
| t0/1 | 80.7% | 50.1% |
| t0/2 | 80.9% | 48.5% |
| t0/3 | 79.7% | 52.4% |
| t1/1 | 78.8% | 47.6% |
| t1/2 | 79.2% | 51.7% |
| t1/3 | 74.6% | 57.1% |
| t2/1 | 73.5% | 45.1% |
| t2/2 | 74.3% | 56.2% |
| t2/3 | 75.3% | 59.3% |
| t3/1 | 73.8% | 66.2% |
| t3/2 | 74.1% | 53.3% |
| t3/3 | 74.0% | 61.4% |
| F1 | 73.8% | 67.4% |
| F2 | 73.7% | 60.7% |
| F3 | 72.9% | 60.9% |
| S1 | 73.0% | 80.1% |
| S2 | 72.9% | 78.6% |
| S3 | 72.7% | 80.9% |

# References


1. Sardari, R. R. *et al.* Evaluation of the production of exopolysaccharides by two strains of the thermophilic bacterium *Rhodothermus marinus*. *Carbohydr. Polym.* **156,** 1–8 (2017).

2. Kavita, K., Singh, V. K., Mishra, A. & Jha, B. Characterisation and anti-biofilm activity of extracellular polymeric substances from *Oceanobacillus iheyensis*. *Carbohydr. Polym.* **101,** 29–35 (2014).

3. Yee, N., Benning, L. G., Phoenix, V. R. & Ferris, F. G. Characterization of metal-cyanobacteria sorption reactions: a combined macroscopic and infrared spectroscopic investigation. *Environ. Sci. Technol.* **38,** 775–782 (Feb. 2004).

4. Derenne, A., Claessens, T., Conus, C. & Goormaghtigh, E. in *Encyclopedia of Biophysics* (ed Roberts, G. C. K.) 1074–1081 (Springer Berlin Heidelberg, Berlin, Heidelberg, 2013).

5. Parks, D. H. *et al.* A standardized bacterial taxonomy based on genome phylogeny substantially revises the tree of life. *Nat. Biotechnol.* **36,** 996–1004 (2018).
