# Supplementary material for: Synergistic biodegradation of aromatic-aliphatic copolyester plastic by a marine microbial consortium
Source: Nat Commun. 2020 Nov 13;11:5790. doi: 10.1038/s41467-020-19583-2 (PMC7666164; doi:10.1038/s41467-020-19583-2)
Supplement: Supplementary file 3 — Reporting Summary [file 41467_2020_19583_MOESM3_ESM.pdf]

nature research

Corresponding author(s): Basak ÖZTÜRK

Last updated by author(s): Oct 12, 2020

Reporting Summary

Nature Research wishes to improve the reproducibility of the work that we publish. This form provides structure for consistency and transparency in reporting. For further information on Nature Research policies, see our Editorial Policies and the Editorial Policy Checklist.

Statistics

For all statistical analyses, confirm that the following items are present in the figure legend, table legend, main text, or Methods section.

n/a	Confirmed	
☐	☒	The exact sample size (*n*) for each experimental group/condition, given as a discrete number and unit of measurement
☐	☒	A statement on whether measurements were taken from distinct samples or whether the same sample was measured repeatedly
☐	☒	The statistical test(s) used AND whether they are one- or two-sided<br>*Only common tests should be described solely by name; describe more complex techniques in the Methods section.*
☒	☐	A description of all covariates tested
☐	☒	A description of any assumptions or corrections, such as tests of normality and adjustment for multiple comparisons
☐	☒	A full description of the statistical parameters including central tendency (e.g. means) or other basic estimates (e.g. regression coefficient) AND variation (e.g. standard deviation) or associated estimates of uncertainty (e.g. confidence intervals)
☐	☒	For null hypothesis testing, the test statistic (e.g. F, t, r) with confidence intervals, effect sizes, degrees of freedom and P value noted<br>*Give P values as exact values whenever suitable.*
☒	☐	For Bayesian analysis, information on the choice of priors and Markov chain Monte Carlo settings
☒	☐	For hierarchical and complex designs, identification of the appropriate level for tests and full reporting of outcomes
☐	☒	Estimates of effect sizes (e.g. Cohen's *d*, Pearson's *r*), indicating how they were calculated

Our web collection on statistics for biologists contains articles on many of the points above.

Software and code

Policy information about availability of computer code

Data collection	TOC-Control L v. 1.06, Spectrum Quant software v. 10.4, SEMSmart v. 5.05, MassHunter GC/MS Acquisition B.07.06.2704 (Agilent) and Maestro 1.5.4.2/3.5 (Gerstel), NovaSeq Control Software v1.6,RTA Version 3.4.4, bcl2fastq v2.20.0.422
Data analysis	Microsoft Excel 2010 v. 14.0.7252.5000, Spectragryph v. 1.2.1,NanoDrop 2000 v. 1.5, INTAS GelDoc v. 0.2.14, Proteome Discoverer v. 1.4, MetaWrap v.1.2, Trim Galore! v. 0.6.4, MetaSpades v. 3.13.0, CONCOCT v. 1.0.0, MaxBin v. 2.2.5, Metabat v. 2.12.1, checkm v. 1.0.12, GTDBtk v. 0.3.2, SortMeRNA v. 2.1b, RSubread v. 1.34.7, DeSEQ2 v. 1.24.0, apeglm v. 1.6.0, ComplexHeatmap v. 2.1.0, Circos Table Viewer v. 0.63-9, ClusterProfiler v. 3.14.3, R v. 3.6.0, ClustalW v. 2.1, BOXSHADE v. 3.2, iTOL v. 5.5., Metabolite detector version 2.2N-2013-01-15, samtools v. 1.7, Prokka v. 1.14, R v. 3.6.0., Geneious v. 2019.1.1, T-Coffee online platform (http://tcoffee.crg.cat/apps/tcoffee/do:regular, last visited 30.09.2020), ggcorrplot package v. 0.1.3.999.

For manuscripts utilizing custom algorithms or software that are central to the research but not yet described in published literature, software must be made available to editors and reviewers. We strongly encourage code deposition in a community repository (e.g. GitHub). See the Nature Research guidelines for submitting code & software for further information.

Data

Policy information about availability of data

All manuscripts must include a data availability statement. This statement should provide the following information, where applicable:

- Accession codes, unique identifiers, or web links for publicly available datasets
- A list of figures that have associated raw data
- A description of any restrictions on data availability

The metagenomic and metatranscriptomic raw data have been deposited at EBI Metagenomics/MGnify: https://www.ebi.ac.uk/ena/browser/view/PRJEB37199, the metaproteomics data at the ProteomeXchange Consortium via PRIDE with the identifier PXD018391: https://www.ebi.ac.uk/pride/archive/projects/PXD018391.

KEGG (https://www.genome.jp/kegg/pathway.html) and Uniprot (https://www.uniprot.org/) databases were used for protein annotation. Source data are provided with this paper. All other data are available from the corresponding author upon request.

Field-specific reporting

Please select the one below that is the best fit for your research. If you are not sure, read the appropriate sections before making your selection.

☒ Life sciences ☐ Behavioural & social sciences ☐ Ecological, evolutionary & environmental sciences

For a reference copy of the document with all sections, see nature.com/documents/nr-reporting-summary-flat.pdf

Life sciences study design

All studies must disclose on these points even when the disclosure is negative.

Sample size	No methods were used to predetermine sample size. For all experiments where statistical analysis was necessary, we used the minimum required number of three.
Data exclusions	No data was excluded.
Replication	All metagenome/transcriptome/proteome experiments were performed in triplicate. The mineralization experiments were performed in triplicates, and repeated three times. The results of all replications were incorporated into the statistical tests and the manuscript.
Randomization	This is not relevant to our study because samples were always taken from a single bacterial mixed culture.
Blinding	Blinding was not applied as the samples were taken from a single mixed culture. All experiments were performed in technical and biological replicates as described in the mansucript, and all results were incorporated into the data analysis. Therefore, blinding was necessary in the study design.

Reporting for specific materials, systems and methods

We require information from authors about some types of materials, experimental systems and methods used in many studies. Here, indicate whether each material, system or method listed is relevant to your study. If you are not sure if a list item applies to your research, read the appropriate section before selecting a response.

Materials & experimental systems

n/a	Involved in the study	
☒	☐	Antibodies
☒	☐	Eukaryotic cell lines
☒	☐	Palaeontology and archaeology
☒	☐	Animals and other organisms
☒	☐	Human research participants
☒	☐	Clinical data
☒	☐	Dual use research of concern

Methods

n/a	Involved in the study	
☒	☐	ChIP-seq
☒	☐	Flow cytometry
☒	☐	MRI-based neuroimaging
